# Nipple aspirate cytology and pathologic parameters predict residual cancer and nodal involvement after excisional breast biopsy

**DOI:** 10.1054/bjoc.2001.2151

**Published:** 2001-12

**Authors:** E R Sauter, H Ehya, A Mammen, Gary Klein

**Affiliations:** Department of Surgery, Thomas Jefferson University; Department of Pathology, Fox Chase Cancer Center; Philadelphia Health Management Corporation, Philadelphia, PA, USA

**Keywords:** nipple aspirate fluid, mastectomy, biomarkers

## Abstract

We previously demonstrated that abnormal nipple aspirate fluid (NAF) cytology predicted residual breast cancer (RC) and tumour size after excisional biopsy (EB), although normal NAF cytology did not exclude RC. Tumour size correlates with the risk of lymph node (LN) metastases. LN metastases provide prognostic information allowing medical and radiation oncologists to determine the need for adjuvant therapy. We hypothesized that pathologic factors known after EB, combined with NAF cytology, would predict with a high degree of accuracy the presence of RC and LN spread. NAF cytology and pathologic parameters: tumour distance from biopsy margins, multifocal and multicentric disease, sub-type of ductal carcinoma in situ (DCIS) or invasive cancer (IC), grade of DCIS or IC, tumour and specimen size, tumour and biopsy cavity location, presence or absence of extensive DCIS, and biopsy scar distance from the nipple were evaluated bivariately and then by logistic regression (LR) for their association with RC and involved LN (≥ 1 (+) LN, useful to determine chemotherapy need, and ≥ 4 (+) LN, useful to determine radiation need to the chest and axilla). Data were analysed using NAF cytology alone, pathologic parameters alone, and NAF cytology and pathologic parameters combined. The combined LR model was superior in predicting residual cancer (94%) to LR models using NAF cytology (36%) or pathologic parameters (75%) alone. When only subjects with normal NAF cytology were evaluated by LR, the model was 92% sensitive in predicting RC. Tumour size and NAF cytology predicted which patients had ≥ 1 (+) LN, whereas tumour and specimen size predicted which patients had ≥ 4 (+) LN. We propose an alogorithm which, if confirmed in a larger study, may allow clinicians to be more selective in their recommendations of re-excision breast biopsy or mastectomy. © 2001 Cancer Research Campaign http://www.bjcancer.com


British Journal of Cancer (2001) 85(12), 1952–1957
© 2001 Cancer Research Campaign
doi: 10.1054/ bjoc.2001.2151, available online at http://www.idealibrary.com on IDEAL

### Nipple aspirate cytology and pathologic parameters predict residual cancer and nodal involvement after excisional breast biopsy

**ER Sauter[1], H Ehya[2], A Mammen[1] and Gary Klein[3]**

[1]Department of Surgery, Thomas Jefferson University; [2]Department of Pathology, Fox Chase Cancer Center; and [3]Philadelphia Health Management Corporation, Philadelphia, PA, USA

**Summary** We previously demonstrated that abnormal nipple aspirate fluid (NAF) cytology predicted residual breast cancer (RC) and tumour size after excisional biopsy (EB), although normal NAF cytology did not exclude RC. Tumour size correlates with the risk of lymph node (LN) metastases. LN metastases provide prognostic information allowing medical and radiation oncologists to determine the need for adjuvant therapy. We hypothesized that pathologic factors known after EB, combined with NAF cytology, would predict with a high degree of accuracy the presence of RC and LN spread. NAF cytology and pathologic parameters: tumour distance from biopsy margins, multifocal and multicentric disease, sub-type of ductal carcinoma in situ (DCIS) or invasive cancer (IC), grade of DCIS or IC, tumour and specimen size, tumour and biopsy cavity location, presence or absence of extensive DCIS, and biopsy scar distance from the nipple were evaluated bivariately and then by logistic regression (LR) for their association with RC and involved LN (≥ 1 (+) LN, useful to determine chemotherapy need, and ≥ 4 (+) LN, useful to determine radiation need to the chest and axilla). Data were analysed using NAF cytology alone, pathologic parameters alone, and NAF cytology and pathologic parameters combined. The combined LR model was superior in predicting residual cancer (94%) to LR models using NAF cytology (36%) or pathologic parameters (75%) alone. When only subjects with normal NAF cytology were evaluated by LR, the model was 92% sensitive in predicting RC. Tumour size and NAF cytology predicted which patients had ≥ 1 (+) LN, whereas tumour and specimen size predicted which patients had ≥ 4 (+) LN. We propose an alogorithm which, if confirmed in a larger study, may allow clinicians to be more selective in their recommendations of re-excision breast biopsy or mastectomy. © 2001 Cancer Research Campaign http://www.bjcancer.com

**Keywords**: nipple aspirate fluid; mastectomy; biomarkers

**Abbreviations**: AH, atypical hyperplasia; DCIS, ductal carcinoma in situ; EB, excisional biopsy; IC, invasive breast cancer; LCIS, lobular carcinoma in situ; LN, lymph node(s); LR, logistic regression; NAF, nipple aspirate fluid; RC, residual breast cancer

Thousands of women undergo re-excision breast biopsy or mastectomy each year to obtain optimal control of their disease, but only approximately half will be found to have residual cancer. We were encouraged by our prior findings which demonstrated that abnormal (atypical or malignant) nipple aspirate fluid (NAF) cytology was 97% accurate in predicting the presence of residual cancer in the breast. All cases with malignant NAF cytology had residual cancer. Nonetheless, most subjects (57/72, 79%) had normal cytology (not atypical or malignant). The presence of normal NAF cytology did not exclude residual cancer, suggesting that NAF cytology was not sufficiently sensitive to be used alone as a predictor of residual cancer in the breast. Prior studies have demonstrated that clinical parameters such as tumour size (Tafra et al, 1993), tumour at the biopsy margin(s), gross multicentricity and extensive DCIS (Harris et al, 1993; Jordan et al, 1998; Lagios, 1992) are predictive of residual breast cancer, although no one marker is sufficiently sensitive nor specific to be used in clinical practice. Tumour size, as well as grade, are correlated with the presence of lymph node metastases (Bland and Copeland, 1998). It was our hypothesis that by combining NAF cytology with pathologic parameters we would increase our ability to predict which women had residual breast cancer and lymph node metastases. If our hypothesis proved correct, it might be possible to be more selective in our determination of who should have additional surgery, as well as adjuvant chemohormonal and radiation therapy. This study would also allow us to determine which of the markers are most predictive of residual breast cancer and nodal spread.

We chose to include those women who had undergone excisional breast biopsy that we evaluated in our last report, so that the best comparison could be made regarding the importance of pathologic parameters over and above NAF cytology. We selected parameters which could be obtained prior to re-excision or mastectomy, so that they would be most applicable to the clinical situation.

Present efforts to evaluate the breast directly either through evaluation of tissue or individual cells have been hindered because the analysis of these specimens generally required an invasive procedure. The adult non-pregnant, non-lactating breast secretes fluid into the breast ductal system. This fluid can be obtained through aspiration of the nipple with a modified breast pump. Nipple aspiration has the attractiveness of quickly, painlessly, and

*Received 13 September 2001*
*Accepted 20 September 2001*

*Correspondence to:* ER Sauter, Department of Surgery, Thomas Jefferson University, 1025 Walnut Street, Suite 605, Philadelphia, PA 19107, USA

non-invasively obtaining breast epithelial cells, the cells at risk for transformation to breast cancer. We are now able to obtain cellular NAF samples (≥ 10 breast epithelial cells on a slide) in the majority of subjects (Sauter et al, 1997). Those samples which contain few or no breast epithelial cells are also informative, for we have also shown that NAF cytology of low cellularity is associated ($p = 0.001$) with a low breast cancer risk (Sauter et al, 1997).

The pathologic parameters chosen for analysis are of proven or suspected importance in predicting whether a woman has residual breast cancer. We previously demonstrated that abnormal NAF cytology is associated with residual breast cancer and tumour size (Sauter et al, 1999). The goal of this study is to determine if NAF cytology combined with pathologic parameters will increase our ability to predict the presence of residual breast cancer and lymph node metastases after excisional biopsy. If we can predict which subjects have residual cancer and/or lymph node metastases, this should prove useful to guide therapy regarding further surgery, chemohormonal and/or radiation therapy.

### MATERIALS AND METHODS

### Subjects

Seventy-two specimens from 70 subjects aged 30–79 (median 52) years were collected for the study between January 1995 and July 1997 after approval of the Institutional Review Board. Each breast had undergone excisional biopsy demonstrating ductal carcinoma in situ (DCIS) or invasive cancer. A mastectomy was performed a median of 22 (range 2–84) days after excisional biopsy, with the exception of three subjects who received neoadjuvant chemotherapy prior to mastectomy. The subject was determined to have residual cancer if either invasive carcinoma (IC) or DCIS was present in the breast, or no residual cancer if neither IC or DCIS was present. (The latter category included subjects whose specimens contained lobular carcinoma in situ (LCIS), atypical hyperplasia (AH), hyperplasia without atypia, or normal breast tissue.)

Excisional biopsy margins were involved in 52/72 (72%), close in 18/72 (25%), or unknown in 2/72 (3%). Pathology records were also reviewed for tumour multifocality (independent tumours in the same breast quadrant) or multicentricity (independent breast tumours in more than one quadrant), type of ductal carcinoma in situ (DCIS) and/or invasive cancer, grade of in situ and/or invasive cancer, tumour and specimen size (we used biopsy cavity size as a surrogate for specimen size, since information on specimen size was not available for all subjects), tumour and biopsy cavity quadrant, the presence or absence of extensive DCIS, distance of the biopsy scar from the nipple, and ER/PR status. The variables included in the final logistic model were based on sample size and predictive strength of the model.

### Aspiration technique

Nipple fluid was aspirated by a trained physician or nurse clinician using a modified breast pump (Sauter et al, 1996). The breast nipple was cleansed with alcohol, the device was placed over the nipple/areolar complex and the plunger of the aspiration device was withdrawn to the 7 ml level and held for 15 s. Fluid in the form of droplets was collected in capillary tubes. The quantity of fluid varied from 1 µl to 200 µl.

If keratin plugs rather than NAF were obtained after suction was completed, the plugs were removed with an alcohol swab and suctioning repeated. On some occasions, this procedure was repeated two or three times to remove the plugs before fluid was obtained. In order to obtain additional fluid, the nipple was gently compressed. One or two additional droplets of fluid often appeared.

### Cytology

#### *A. Specimen preparation*

The NAF was collected in 50 µl capillary tubes and rinsed into a container with 1 ml of 3% polyethylene glycol in ethanol/isopropanol. The specimen was then cytocentrifuged onto 10 glass slides. Three of the slides were used for cytologic examination. If the slides contained < 10 epithelial cells, two additional slides were examined. The remaining slides (5 or 7) were stored for biomarker studies. The slides selected for cytologic examination were washed twice in 95% ethanol for 10 min each, rehydrated in tap water and stained by the Papanicolaou method.

#### *B. Specimen interpretation*

The Papanicolaou-stained smears were examined by a cytopathologist (HE) experienced with breast cytology. The examiner was not aware of the histopathologic findings of each specimen. Moreover, since the NAF specimens analysed in this study were mixed with specimens from subjects with an intact breast, the examiner was not aware of which NAF specimens were from mastectomies. Each specimen was designated as containing scant epithelial cells (Class I), normal epithelial cells (Class IIA), hyperplasia without atypia (Class IIB), atypia (Class III), or malignant cells (Class IV), using criteria previously described (Sauter et al, 1997). This is a modification of terminology used by King et al (1983), and is to be differentiated from the Papanicolaou classification.

### Histology

#### *A. Specimen preparation*

The breast specimen was fixed in 10% neutral buffered formalin for 16–24 h and embedded in paraffin wax. Eighteen to 20 representative blocks were prepared, and one 4 µm slide per block was cut and stained with haematoxylin and eosin (H&E).

#### *B. Specimen interpretation*

Histologic review of each H&E slide from the excisional biopsy and mastectomy was performed. Margin status was assessed in the excisional biopsies. A margin was defined as positive if tumour was present at the margin, close if tumour was present ≤ 2 mm from the margin, and negative if present > 2 mm from the margin.

### Statistical analysis

Bivariate analyses were performed between biomarker characteristics and residual cancer in the breast, the presence of one or more positive lymph nodes and the presence of four or more positive lymph nodes (Table 1). To test for significance, the difference of proportions *t*-test was employed for dichotomous independent variables (Brown and Hollander, 1977; Jordan et al, 1998). For independent variables with three or more ordered categories, Somer's D (asymmetric extension of gamma statistic) was applied,

*© 2001 Cancer Research Campaign*

**Table 1** Bivariate analysis of biomarker characteristics by residual disease in the breast[1] and by lymph node status

| | Residual breast cancer | | Positive lymph nodes | | | |
|---|---|---|---|---|---|---|
| | | | 1 or more | | 4 or more | |
| | Yes | No | Yes | No | Yes | No |
| Cytology ($n$ = 72) | | | | | | |
| Normal | 25*** | 32 | 19* | 38 | 7 | 50 |
| Atypical or malignant | 14 | 1 | 11 | 4 | 3 | 12 |
| Tumour characteristics ($n$ = 71) | | | | | | |
| Multifocal disease | 15 | 9 | 8 | 16 | 2 | 22 |
| Multicentric disease | 5 | 1 | 2 | 4 | 0 | 6 |
| Neither | 19 | 22 | 19 | 22 | 7 | 34 |
| Specimen size in cm ($n$ = 72) | | | | | | |
| < 1 | 12 | 4 | 9 | 7 | 1 | 15 |
| 1–1.9 | 11 | 8 | 6 | 13 | 2 | 17 |
| 2–2.9 | 3 | 7 | 3 | 7 | 2 | 8 |
| 3–3.9 | 9 | 10 | 7 | 12 | 2 | 16 |
| 4–4.9 | 2 | 1 | 1 | 2 | 1 | 2 |
| ≥ 5 | 2 | 3 | 4 | 1 | 2 | 4 |
| Biopsy margins ($n$ = 67) | | | | | | |
| Negative/close | 7** | 16 | 7 | 16 | 2 | 21 |
| Positive | 29 | 15 | 19 | 25 | 6 | 38 |
| Primary tumour size in cm ($n$ = 71) | | | | | | |
| in situ | 7* | 8 | 1*** | 14 | 0* | 15 |
| < 2 | 11 | 16 | 6 | 21 | 3 | 24 |
| 2–4.9 | 12 | 6 | 13 | 5 | 3 | 15 |
| ≥ 5 | 6 | 2 | 6 | 2 | 3 | 5 |
| invades other tissues | 3 | 0 | 3 | 0 | 0 | 3 |
| Tumor grade ($n$ = 65) | | | | | | |
| 1 | 3 | 5 | 1** | 7 | 0# | 8 |
| 2 | 15 | 12 | 9 | 18 | 3 | 24 |
| 3 | 18 | 12 | 18 | 12 | 6 | 24 |
| Scar distance from the nipple in cm ($n$ = 50) | | | | | | |
| < 1 | 2 | 0 | 1 | 1 | 0 | 2 |
| 1–2.9 | 3 | 5 | 3 | 5 | 0 | 8 |
| 3–4.9 | 11 | 10 | 5 | 16 | 3 | 18 |
| 5+ | 11 | 8 | 8 | 11 | 3 | 16 |
| Extensive DCIS ($n$ = 64) | | | | | | |
| No | 20 | 24 | 20 | 24 | 9 | 35 |
| Yes | 14 | 6 | 8 | 12 | 0 | 20 |
| Estrogen receptor ($n$ = 53) | | | | | | |
| Negative | 5 | 8 | 7 | 6 | 3 | 10 |
| Positive | 24 | 16 | 19 | 21 | 6 | 34 |
| Progesterone receptor ($n$ = 52) | | | | | | |
| Negative | 15 | 10 | 14 | 11 | 5 | 20 |
| Positive | 14 | 13 | 12 | 15 | 4 | 23 |

[1]The bivariate relationships between surgery result (residual cancer present or absent) and cytology, biopsy margins, nipple involvement, EIC, ER and PR were tested using the Difference of Proportions test (Brown and Hollander, 1977; Jordan et al, 1998). The bivariate relationships between surgery result and specimen size, primary tumour size, residual tumour size, tumour characteristics, tumour grade and scar distance from the nipple were tested using the Somer's D statistic for ordered categories (Everitt, 1977).
***$P$ < 0.001, **$P$ < 0.01, *$P$ < 0.05, #$P$ < 0.10

which is preferential to other non-parametric tests such as Mann-Whitney and Kruskall-Wallis in situations where there are a limited number of ordered categories (Everitt, 1977).

Stepwise logistic regression models (Agresti, 1990) were fit to the data to determine whether residual cancer in the breast was associated with cytologic grouping and pathologic parameters for which data were available the vast majority of subjects and on bivariate analysis contributed to the model. The pathologic parameters included specimen margins, specimen size, presence of multifocality and/or multicentricity, and tumour size. The same pathologic parameters were used to determine the best model to predict the presence of lymph node status: either 0 vs 1 or more positive lymph nodes, or 0–3 vs 4 or more positive lymph nodes. Initially we evaluated all subjects, and then an important subgroup, those with normal cytology (no atypia or carcinoma).

### RESULTS

We first determined the bivariate relationships between NAF cytology and pathologic parameters with the presence of residual breast cancer and lymph node involvement (Table 1). Abnormal NAF cytology ($P$ < 0.001), positive biopsy margins ($P$ < 0.01) and

*© 2001 Cancer Research Campaign*

large primary tumour size (*P* < 0.05) were associated with the presence of residual breast cancer. Large primary tumour size (*P* < 0.001), high tumour grade (*P* < 0.01) and abnormal NAF cytology (*P* < 0.05) were associated with the presence of ≥ 1 positive lymph nodes, whereas only large primary tumour size (*P* < 0.05) and tumour grade (*P* < 0.10) were associated with ≥ 4 positive lymph nodes.

Taking into consideration both sample size and bivariate relationships, cytology, tumour size, the presence of multifocality and/or multicentricity, specimen size, and margin status were considered in our final logistic regression model. Forward selection stepwise logistic regression was first implemented on the entire data set. In a prior publication we demonstrated that subjects with abnormal (atypical or malignant) NAF cytology after excisional biopsy were very likely to have residual breast cancer. Next, we evaluated pathologic parameters which could help predict the presence of residual breast cancer in subjects with normal NAF cytology. In the latter group, NAF cytology was of no help in predicting residual breast cancer. The four pathologic parameters: presence of multifocality and/or multicentricity, tumour size, specimen size and margin status led to a model (Table 2) which was highly sensitive (92%) and specific (80%) in predicting residual breast cancer ($R^2$ = 0.460).

For our entire data set (Table 2), the partial effects of NAF cytology (*P* < 0.001), margin status and presence of multifocality and/or multicentricity (*P* < 0.01 for both), and specimen size (*P* < 0.05) were found to be significantly associated with the risk of residual cancer. Tumour size was not significant in the regression model mainly because of its shared correlation with margin status, which is a better predictor of residual breast cancer. The resulting four markers yielded a highly predictive model ($R^2$ = 0.593) for residual breast cancer. In this way, we increased the sensitivity of non-invasive methods to predict residual breast cancer from 36% using NAF cytology alone to 94% using all four markers. NAF cytology proved to be an important addition to pathologic factors, for when information for NAF cytology was not available, the predictive value ($R^2$ = 0.294 vs 0.593) and the sensitivity of the model declined (75% vs 94%).

We used the same parameters in our models of all subjects, and those with normal cytology, to predict which subjects had involved lymph nodes (Table 3). When subjects with normal cytology were evaluated, only tumour size was significantly predictive of the presence of both one or more positive nodes (*P* < 0.001) as well as four or more positive nodes (*P* < 0.05). For all subjects, tumour size (*P* < 0.001) and cytology (*P* < 0.05) were significant in the model ($R^2$ = 0.475) to determine which subjects had one or more

**Table 2** Stepwise logistic regression results for all specimens in the model of residual breast cancer[1]

| Dependent (independent) Variables | Nagelkerke $R^2$ | Sensitivity (%) | Specificity (%) | PPV (%) | NPV (%) |
|---|---|---|---|---|---|
| Normal cytology (margins**, mf/mc[2,] *, specimen size* and tumour size[NS]) | 0.474 | 22/24 (92) | 24/30 (80) | 22/28 (79) | 24/26 (92) |
| All subjects (cytology***, margins**, mf/mc**, specimen size* and tumour size[NS]) | 0.593 | 34/36 (94) | 24/31 (77) | 34/41 (83) | 24/26 (92) |

[1]Complete data were available for 54 of 57 subjects with normal cytology and 67 of 72 total subjects. [2]mf/mc: Presence of either multifocal or multicentric disease. Two additional independent variables were examined in separate models but not included because they decreased the sample to < 60 and had only minor impact. ****P* < 0.001, ***P* < 0.01, **P* < 0.05, [#]*P* < 0.10, [NS] not significant.

**Table 3** Stepwise logistic regression results for number of lymph nodes involved with cancer[1]

| Dependent (independent) variables | Nagelkerke $R^2$ | Sensitivity (%) | Specificity (%) | PPV (%) | NPV (%) |
|---|---|---|---|---|---|
| Normal cytology | | | | | |
| zero vs one or more (tumour size***, mf/mc[2, NS], specimen size[NS] and biopsy margins[NS]) | 0.344 | 12/17 (71) | 30/37 (81) | 12/19 (63) | 30/35 (86) |
| 0–3 vs 4 or more (tumour size*, specimen size[NS], mf/mc[NS] and biopsy margins[NS]) | 0.157 | 0/6 (0) | 48/48 (100) | 0/0 (–) | 48/54 (89) |
| All subjects | | | | | |
| zero vs one or more (tumour size***, cytology*, specimen size[NS], mf/mc[NS] and biopsy margins[NS]) | 0.475 | 20/26 (77) | 33/41 (81) | 20/28 (71) | 33/39 (85) |
| 0–3 vs, 4 or more (tumour size*, specimen size[#] and cytology[NS], mf/mc[NS] and biopsy margins[NS]) | 0.207 | 1/8 (13) | 59/59 (100) | 1/1 (100) | 59/66 (89) |

[1]Complete data was available for 54 of 57 subjects with normal cytology. [2]mf/mc: Presence of either multifocal or multicentric disease. Due to the limited sample size, a moderate to strong predictive relationship is required for statistical significance. ****P* < 0.001, ***P* < 0.01, **P* < 0.05, [#]*P* < 0.10, [NS] not significant.

© 2001 Cancer Research Campaign

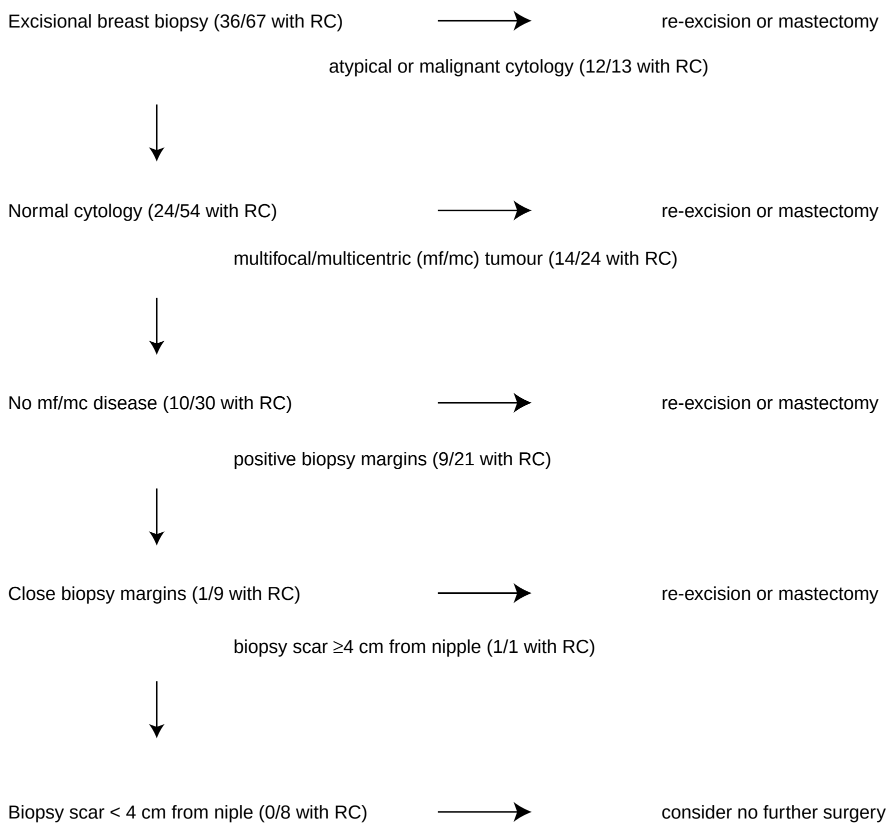

**Figure 1** Need for re-excision breast biopsy or mastectomy. Algorithm to determine which subjects have residual breast cancer (RC). This is based on a limited sample set and must be confirmed before being used in clinical practice to determine the need for re-excision breast biopsy or mastectomy.

positive lymph nodes, while tumour size ($P < 0.05$) and specimen size ($P < 0.10$) were significant in predicting which subjects had four or more positive lymph nodes.

Our logistic regression model considers NAF cytology and pathologic parameters together in order to determine the presence of residual breast cancer or lymph node spread. An alternative way to look at the predictors is by developing a decision tree, or algorithm (Figure 1). Using this strategy, the combination of normal cytology, absence of multifocality or multicentricity, close biopsy margins and biopsy scar < 4 cm from the nipple defined a population of women who were all free of residual breast cancer. These eight women comprise 26% (8/31) of the women in the model who did not have residual disease. We attempted to develop an algorithm which defined subjects in our data set who had negative lymph nodes, but it was not sufficiently discriminative (data not shown).

### DISCUSSION

When we initiated this study, we wished to identify markers in NAF which were associated with residual cancer in the breast and disease spread to lymph nodes. In our prior report, we evaluated subjects diagnosed by either needle or excisional biopsy. However, since 100% of the mastectomies performed after needle biopsy alone had residual DCIS or invasive cancer, in this study we chose to focus on subjects with presumed residual disease after excisional biopsy, in which only 39/72 (54%) mastectomies performed contained residual DCIS or invasive cancer.

After excisional biopsy, many women undergo mastectomy for presumed residual cancer. Thousands of women who undergo mastectomy after excisional biopsy will not have in situ or invasive cancer found in the mastectomy specimen. The chance of finding residual cancer is related to tumour margin status in the diagnostic specimen. If the margin is close, the likelihood is 23–32%; if positive, 53–65%; and if unknown, approximately 45% (Frazier et al, 1989; Gwin et al, 1993). In our prior publication, we reported that NAF cytology (normal vs atypical or malignant) was 97% specific but only 36% sensitive in predicting the presence of residual cancer, with the only false positive coming from a specimen demonstrating atypia.

The breast ducts of adult non-pregnant women secrete small amounts of fluid (Keynes, 1923). This fluid does not escape because the nipple ducts are occluded by smooth muscle contraction, dried secretions, and keratinized epithelial plugs. Breast fluid can be obtained by nipple aspiration in a significant proportion of women without spontaneous nipple discharge with the use of a modified breast pump (Petrakis et al, 1975). This fluid contains several types of cells, including exfoliated breast epithelial cells (King et al, 1975). Because breast cancer develops from ductal and lobular epithelium, NAF is a potentially useful epidemiologic and clinical research tool.

Wrensch et al (1992) evaluated NAF in a cohort of subjects with normal breast cancer risk. In this population, they demonstrated that subjects with NAF which contained normal cytology, hyperplasia without atypia, or atypical hyperplasia have a risk of breast cancer similar to subjects who have a biopsy with a similar diagnosis. We found (Sauter et al, 1999) that malignant cells in NAF after excisional biopsy were 100% and atypical cells 88% predictive of the presence of residual breast cancer.

 *© 2001 Cancer Research Campaign*

Since NAF cytology is only 36% sensitive in detecting residual cancer, a finding of abnormal NAF cytology is highly suggestive of the presence of residual cancer, but normal cytology does not exclude the possibility that residual cancer exists. We therefore evaluated pathologic markers known to be associated with breast cancer, to determine if we could increase our ability to predict the presence of residual disease.

NAF cytology, the presence of multifocality and/or multicentricity, specimen size, and biopsy margins were the characteristics which helped to create the best model to predict the presence of residual breast cancer. Using this combination of markers, we were able to increase our sensitivity from 36% to 94%. NAF cytology is an important predictor of residual cancer, for without it the association drops from $R^2 = 0.59$ to $R^2 = 0.29$, and the sensitivity decreases from 94% to 75%. For subjects with normal cytology, the model is 92% sensitive in predicting the presence of residual breast cancer. This dramatic increase in sensitivity resulted in a decrease in specificity of 15–20% compared to the examination of NAF cytology alone.

We developed an algorithm (Figure 1) which correctly identified 26% of women who did not have residual breast cancer. These findings are preliminary and certainly need to be reproduced in a larger multi-institutional study. If confirmed, the algorithm may be useful in helping clinicians to determine who should and should not undergo re-excision breast biopsy or mastectomy.

We found that both NAF cytology and pathologic parameters contributed to the model which best predicted the presence of residual breast cancer and nodal spread, and that the combination of parameters was both highly sensitive and specific in predicting residual cancer and moderately sensitive but highly specific in predicting nodal spread. These findings provide insight into breast cancer tumour development and progression, and may prove useful in guiding therapy.

### ACKNOWLEDGEMENT

This work was supported, in part, by NIH grant CA 87391.

### REFERENCES

Agresti A (1990) *Categorical data analysis*. Wiley & Sons: New York.

Bland KI and Copeland EA (1998) *The breast: comprehensive management of benign and malignant diseases.* WB Saunders: Philadelphia

Brown BW and Hollander M (1977) *Statistics: A Biomedical Introduction*. Wiley & Sons: New York

Everitt BS (1977) *The Analysis of Contingency Tables.* Chapman & Hall: London

Frazier TG, Wong RW and Rose D (1989) Implications of accurate pathologic margins in the treatment of primary breast cancer. *Arch Surg* **124**: 37–38

Gwin JL, Eisenberg BL, Hoffman JP, Ottery FD, Boraas M and Solin LJ (1993) Incidence of gross and microscopic carcinoma in specimens from patientswith breast cancer after re-excision lumpectomy. *Ann Surg* **218**: 729–734

Harris JR, Morrow M and Bonnadonna G (1993) *Cancer of the breast*. In: *Cancer: Principles and Practice of Oncology.* JB Lippincott: Philadelphia

Jordan K, Ong BN and Croft P (1998) *Mastering Statistics: A Guide for Health Service Professionals and Researchers*. Stanley Thornes, Ltd: Cheltenham, UK

Keynes G (1923) Chronic mastitis. *Br J Surg* **11**: 89–121

King EB, Barrett D, King MC and Petrakis NL (1975) Cellular composition of the nipple aspirate specimen of breast fluid. I. The benign cells. *Am J Clin Pathol* **64**: 728–738

King EB, Chew KL, Petrakis NL and Ernster VL (1983) Nipple aspirate cytology for the study of breast cancer precursors. *J Natl Cancer Inst* **71**: 1115–1121

Lagios MD (1992) Pathologic features related to local recurrence following lumpectomy and irradiation. *Semin Surg Oncol* **8**: 122–128

Petrakis NL, Mason L, Lee R, Sugimoto B, Pawson, S and Catchpool F (1975) Association of race, age, menopausal status, and cerumen type with breast fluid secretion in nonlactating women, as determined by nepple aspiration. *J Natl Cancer Inst* **54**: 829–834

Sauter ER, Daly M, Linahan K, Ehya H, Engstrom PF, Bonney G, Ross EA, Yu H and Diamandis E (1996) Prostate-specific antigen levels in nipple aspirate fluid correlate with breast cancer risk. *Cancer Epidemiol Biomarkers Prev* **5**: 967–970

Sauter ER, Ross E, Daly M, Klein-Szanto A, Engstrom PF, Sorling A, Malick J and Ehya H (1997) Nipple aspirate fluid: a promising non-invasive method to identify cellular markers of breast cancer risk. *Br J Cancer* **76**: 494–501

Sauter ER, Ehya H, Babb J, Diamandis E, Daly M, Klein-Szanto A, Sigurdson E, Hoffman J, Malick J and Engstrom PF (1999) Biological markers of risk in nipple aspirate fluid are associated with residual cancer and tumour size. *Br J Cancer* **81**: 1222–1227

Tafra L, Guenther JM and Giuliano AE (1993) Planned segmentectomy. A necessity for breast carcinoma. *Arch Surg* **128**: 1014–1018; discussion 1018–1020

Wrensch MR, Petrakis NL, King EB, Miike R, Mason L, Chew KL, Lee MM, Ernster VL, Hilton JF and Schweitzer R (1992) Breast cancer incidence in women with abnormal cytology in nipple aspirates of breast fluid. *Am J Epidemiol* **135**: 130–141

*© 2001 Cancer Research Campaign*
